# Molecular Characterization and Genetic Diversity of Ginkgo (*Ginkgo biloba* L.) Based on Insertions and Deletions (InDel) Markers

**DOI:** 10.3390/plants12132567

**Published:** 2023-07-06

**Authors:** Dan Wang, Qi Zhou, Linlin Le, Fangfang Fu, Guibin Wang, Fuliang Cao, Xiaoming Yang

**Affiliations:** Co-Innovation Center for Sustainable Forestry in Southern China, Nanjing Forestry University, Nanjing 210037, China; wandan0628@njfu.edu.cn (D.W.); zhouqi@njfu.edu.cn (Q.Z.); lll@njfu.edu.cn (L.L.); fffu@njfu.edu.cn (F.F.); gbwang@njfu.com.cn (G.W.); caofl@njfu.edu.cn (F.C.)

**Keywords:** Gingko, InDel marker, genetic diversity, core collections

## Abstract

As a “living fossil”, ginkgo (*Ginkgo biloba* L.) has significant ornamental, medicinal, and timber value. However, the breeding improvement of ginkgo was limited by the lack of enough excellent germplasms and suitable molecular markers. Here, we characterized numerous polymorphic insertion/deletion (InDel) markers using RAD-seq in 12 different ginkgo cultivars. The total of 279,534 InDels identified were unequally distributed across 12 chromosomes in the ginkgo genome. Of these, 52.56% (146,919) and 47.44% (132,615) were attributed to insertions and deletions, respectively. After random selection and validation, 26 pairs of polymorphic primers were used for molecular diversity analysis in 87 ginkgo cultivars and clones. The average values of observed heterozygosity and polymorphism information were 0.625 and 0.517, respectively. The results of population structure analyses were similar to those of neighbor-joining and principal component analyses, which divided all germplasms into two distinct groups. Moreover, 11 ginkgo core collections accounted for approximately 12.64% of the total ginkgo germplasms obtained, representing well the allelic diversity of all original germplasms. Therefore, these InDels can be used for germplasm management and genetic diversity analyses in ginkgo and the core collections will be used effectively for ginkgo genetic improvement.

## 1. Introduction

Ginkgo (*Gingko biloba* L.) is a well-known living gymnosperm fossil dating back to at least 200 million years ago [1,2]. Ginkgo has been used as herbal medicine for thousands of years given its high content of effective pharmacological components including terpenoids and flavonoids [3]. The standardized extracts (EGb761) of ginkgo leaves containing 24% flavonol glycosides and 6% terpene lactones, along with ginkgolic acids and other constituents, are considered a drug or dietary supplement in many countries [4,5]. Given its efficacy and pharmacological activity, ginkgo has been used to treat cardiovascular, cerebrovascular, and Alzheimer’s diseases [6]. Moreover, in the herbal remedy market, ginkgo is of great economic value, being a top-selling dietary supplement [5]. Currently, ginkgo breeding focuses on increasing the production of specific secondary metabolic products with nutritive or pharmacological functions. Despite collections and cultivations of ginkgo cultivars and clones, there is considerable synonymy, homonymy, and genetic redundancy with these germplasms, which severely hinders the progress of ginkgo breeding.

Fruit tree breeding largely depends on excellent and diverse germplasms [7]. The core germplasm collection includes a subset of germplasms that comprise the highest genetic diversity and least repeatability of core species. Core germplasm as a useful strategy has been successfully used to lessen the impact of the redundancy in germplasm resources that lowers management and conservation effectiveness [8]. Generally, tree germplasms were unusually collected from natural populations and underwent long generation times but with brief domestication history. Therefore, there was a relatively high intrinsic genetic variability among different wood plants that has a relatively high intrinsic genetic diversity and core germplasm collections represent 10–45% of the total germplasms obtained from these species [9,10]. Molecular markers, as powerful and inexpensive tools, have been widely used for genotypic fingerprinting, analyzing marker-assisted breeding, genetic diversity, phylogenetic analysis, as well as establishing core germplasm collections [11,12]. In addition, marker-assisted selection has been widely applied to perennial tree breeding, significantly increasing the selection efficiency [13]. Meanwhile, the genetic diversity of ginkgo has been investigated by multiple molecular markers such as simple sequence repeat (SSR) [14], amplified fragment length polymorphism (AFLP) [15], and single nucleotide polymorphism [16]. However, previous research mainly focused on exploring molecular diversity and population structure in wide or semi-wide ginkgo germplasms. Due to the lack of excellent ginkgo cultivars and clones, a reference for further use of germplasm resources is now unavailable. Currently, core germplasms are used to capture allelic variations and represent the species diversity in multiple annual crops but only in a few perennial trees due to the difficulty of wood plant breeding [17,18]. In ginkgo, however, few reports focused on core collections based on morphological or genetic information. A comprehensive understanding of the genetic variation of common ginkgo cultivars and clones is immediately required to promote ginkgo breeding. 

Insertion-deletions (InDels) were a common source of variation widely distributed throughout the genome and successfully identified in diverse annuals or perennial plant species, such as Arabidopsis [19], rice [20], chickpea [12], common bean [21], poplar [22], and citrus [23]. InDels arise from errors in sequence replication, insertion of transposable elements, or unequal crossover events [24]. InDels are suitable for genetic analysis primarily due to inherent genetic attributes such as codominant inheritance and multi-allelic and wide genome distribution [12]. With its low cost and high throughput, restriction site-associated DNA sequencing (RAD-seq) can significantly reduce genome complexity and identify abundant InDel makers with or without a reference genome [25]. The increased availability of genome data has allowed the development of a large number of InDels in many species using RAD-seq [26]. Nevertheless, to our knowledge, just a few InDels have been used for genetic diversity assessment and molecular marker-assisted breeding in ginkgo.

Thus, this study aimed to (1) characterize InDel variations in the ginkgo genome based on the RAD-seq and develop polymorphic InDel markers; (2) analyze the molecular diversity of different ginkgo cultivars and clones; and (3) screen core germplasms to promote the process of ginkgo improvement in the future. This research will lay a solid foundation for the conservation, characterization, and utilization of ginkgo germplasms.

## 2. Results

### 2.1. Sequencing Data Quality and Processing 

To identify variations in the ginkgo genome, 12 different ginkgo cultivars were selected for RAD sequencing. A total of 127.37 Gb of clean reads were generated with a size ranging from 8.08 to 14.61 Gb, with an average of 10.61 Gb per sample (Table S2). In addition, all samples were free from contamination and our sequencing data revealed a relatively high sequence Phred quality score (raw Q30 > 90%, clean Q30 > 90%), with a stable GC content of 40.60–54.17%, lower than the AT content (Table S2). Sequence data showed an average genome coverage of 1.7% and an average mapping rate of 87.46% (Table S2). Nearly all reads could be mapped to 12 different chromosomes in the ginkgo genome (Figure S1).

### 2.2. InDel Characteristics in the Ginkgo Genome 

**Figure 1:**
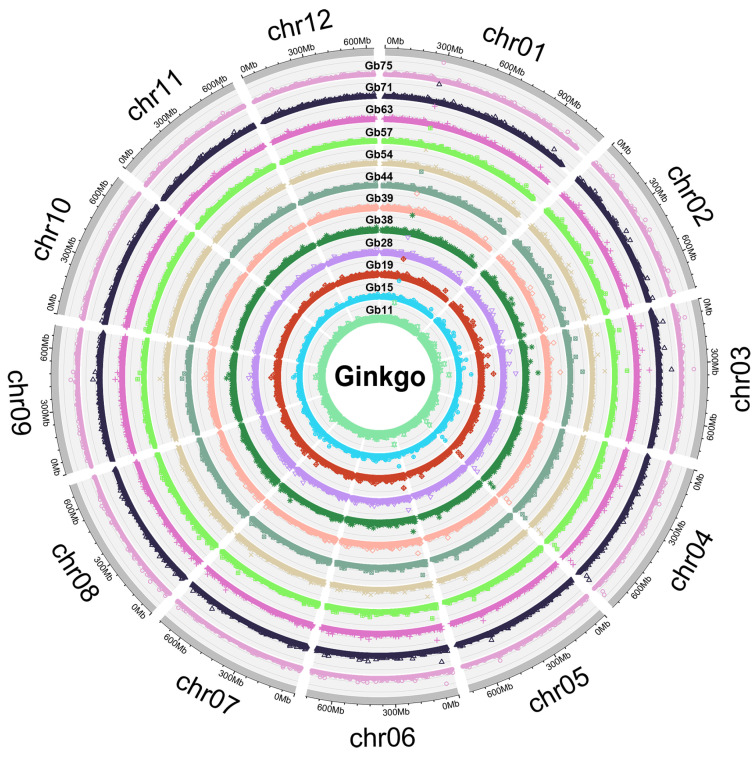
Circular representation of the distribution of Indel markers density in 12 different chromosomes based on the RAD-seq with 12 different ginkgo cultivars (Gb11, Gb15, Gb19, Gb28, Gb38, Gb39, Gb44, Gb54, Gb57, Gb63, Gb71, and Gb75).

**Figure 2:**
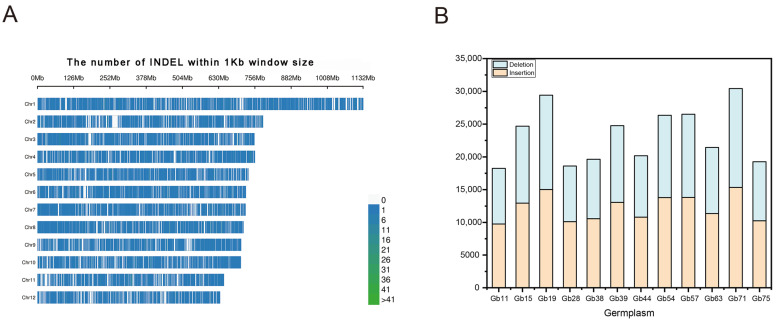
The distribution and characteristics of Indels in ginkgo. The patterns of Indel markers distributed on each chromosome in ginkgo (**A**); Comparative distribution of the insertions and deletions among 12 ginkgo cultivars (**B**).

In this study, we focused on small, predominant InDel fragments (2–10 bp) to avoid the high InDel error rates associated with longer InDels [27]. A total of 279,534 InDel markers were identified by comparative analyses of raw sequences of 12 different ginkgo cultivars with strict filter parameters and the distribution of InDels in each species was different (Figure 1). There were 27.30 InDels per megabase and all InDels were unequally distributed across 12 chromosomes in the ginkgo genome (Figure 2A). Of these, 52.56% (146,919) and 47.44% (132,615) were attributed to insertions and deletions, respectively (Figure 2B). All identified InDels were contributed by homozygosity (143,146, 51.21%) and heterozygosity (136,388, 48.79%) and these two typical InDels took up similar proportions in 12 different ginkgo cultivars. Except for two cultivars (Gb11 and Gb32; Table S1), where homologous InDels accounted for a relatively low proportion (less than 45%), the rest had more homologous InDels (Figure S2). Two bases in InDel loci accounted for 35.10% (98,117) of the total InDels and were found to be the majority of InDel loci. The number of InDel loci significantly decreased with increasing in InDel length. Accordingly, the length of InDel loci showed a highly significant and negative correlation with the number of InDel loci (r = −0.045, *p* = 0.008). Over 2.08% (5814) of the total number of InDels was found in coding regions, followed by 21.91% (61,245) in introns and 76.01% (212,475) in intergenic regions. Unique InDels across these 12 different ginkgo cultivars were also identified and the range of the number of InDels varied significantly. There was a total of 99,097 (35.45%) unique InDels across these 12 different cultivars. Among individual cultivars, the maximum number of InDels was founded in ginkgo (Gb70; Table S1) with 15,728 (15.87%), whereas the minimum one was founded in ginkgo (Gb32; Table S1) with 3378 (3.41%). Interestingly, there were 452 insertions and 284 deletions that appeared simultaneously in at least 6 of the 12 different ginkgo cultivars (Table S3). 

### 2.3. Experimental Validation of InDel Polymorphisms

**Figure 3:**
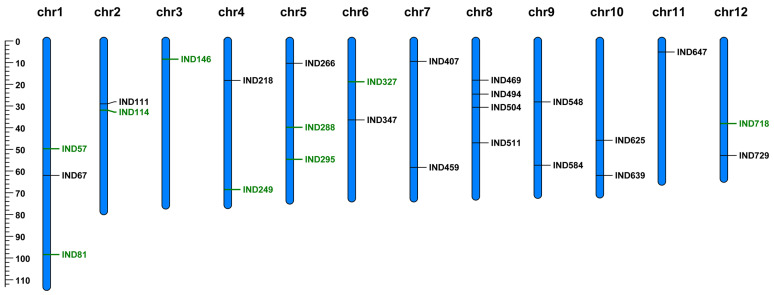
Comparative distribution of Indel markers among 12 ginkgo cultivars. Insertion and deletion markers were in green and black font, respectively.

The site-specific or single-copy primer pairs covering InDels were considered potentially useful InDels. A total of 100 pairs of InDel primers distributed unevenly on 12 chromosomes on all cultivars were selected randomly to validate polymorphisms. These markers were used for amplification in the same 12 ginkgo cultivars used for RAD-seq. As a result, 26 dimorphic InDel markers produced the expected band size and showed polymorphisms, regarded as the best-scored dimorphic markers for further analyses (Figure 3 and Table S4). Furtherly, based on the annotation analysis, 80.76% (21/26) of the developed polymorphic InDels appeared in coding sequence regions with potential functions associated to protein kinases or hormone metabolism in ginkgo (Table S4).

### 2.4. Genetic Diversity and Population Structure Analysis

There were 107 alleles detected by 26 polymorphic InDel markers, with an average of 4.115 alleles per site, among which 6 alleles were detected at the IND57 locus. The alleles of 12 different loci (IND67, IND81, IND218, IND288, IND295, IND347, IND511, IND548, IND584, IND625, IND647, and IND718) were the same, having 4 alleles. Across the 26 loci, the number of Ne varied from 2.094 to 5.810, with an average of 3.612. The average of He and Ho were 0.499 and 0.625, respectively. The average PIC was 0.517, suggesting most InDel loci showed a relatively high level of polymorphism. Accordingly, the InDel locus (IND57) had the highest genetic diversity, whereas the InDel locus (IND114) had the lowest. All these genetic parameters are summarized in Table 1.

**Figure 4:**
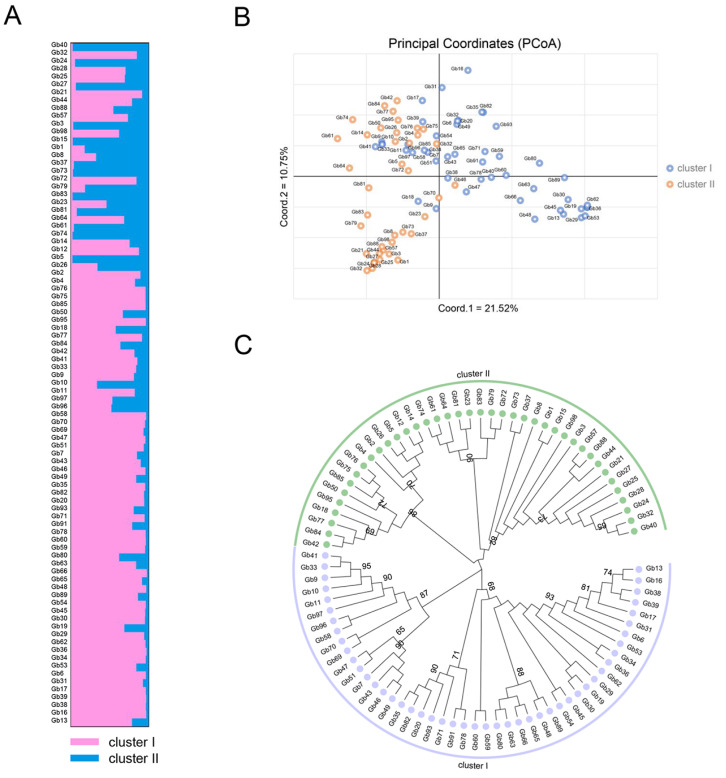
Genetic clustering of 87 ginkgo cultivars and clones with 26 InDel markers. (**A**) Genetic structure results based on the Bayesian clustering model at K = 2. Cluster I and cluster II are presented in pink and blue color, respectively; (**B**) Principal component analysis (PCoA); (**C**) Phylogenetic tree based on the genetic distance.

The population structure analysis illustrated that the K value increased continuously with increasing LnP(D) value. The Δ*K* analysis revealed a sharp Δ*K* peak at K = 2, indicating two genetically distinct sub-populations (Figure S3). Based on the membership coefficient criterion (0.75), each ginkgo germplasm was assigned to a certain population. There were 47 and 40 ginkgo germplasms grouped in cluster I and cluster II, respectively (Figure 4A). Some germplasms belonged to four major groups (“Changzi”, “Fozhi”, “Zhongzi”, and “Yuanzi”) and showed mixed distribution in cluster I and II. Interestingly, the ginkgo germplasms belonging to the “Yuanzi” group were grouped together, showing a distinct clustering rule from others. Moreover, ginkgo germplasms with the same geographical origins appeared in the same cluster. For example, three genotypes (Gb46, Gb47, and Gb47; Table S1) and three genotypes (Gb58, Gb59, and Gb60; Table S1) were selected from Zheng’an county (Guizhou, China) and Changxin county (Zhejiang, China), respectively, grouped in cluster I. Similarly, five genotypes (Gb72, Gb73, Gb74, Gb75, and Gb76; Table S1) from Anlu city (Hubei, China) and four genotypes (Gb26, Gb27, Gb28, and Gb29; Table S1) from Pizhou city (Jiangsu, China) by selective breeding were grouped in cluster II. 

To better characterize the genetic variation among different ginkgo germplasms, we performed PCoA analysis based on Nei’s unbiased genetic distance (Figure 4B). The scatter plot generated from PCoA clustered the 87 genotypes of ginkgo into two groups based on similarity indices. The first, second, and third coordinates accounted for 21.51%, 10.75%, and 7.96% variation, respectively, together accounting for a total cumulative variation of 40.22%. We further explored the genetic differentiation and relationships among different ginkgo germplasms based on the NJ tree (Figure 4C). Similar to the PCoA and STRUCTURE analysis, the NJ dendrogram confirmed the existence of two clusters, congruent with the model-based population structure and PCoA analysis. 

### 2.5. Establishment and Evaluation of the Ginkgo Core Collections

**Figure 5:**
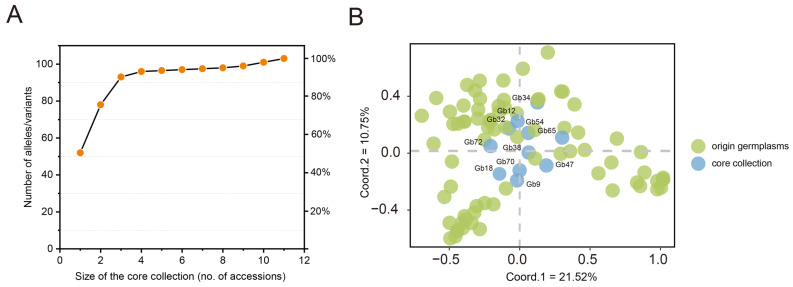
The genetic diversity of core ginkgo collections. Identification of the core collections of ginkgo germplasms based on the strategy of maximizing allelic diversity (**A**). Principle coordinates analysis (PCoA) of constructed core collections and the original germplasms (**B**).

A core collection of ginkgo germplasm was constructed to reflect all the genetic diversity discovered in this study considering that the smallest core collections could represent the whole diversity detected based on 26 InDel markers (Figure 5A). Based on the maximizing strategy, the number of sampled alleles increased fast with the expanding sample size. However, for a sample size of 11 individuals, the curve gradually levelled out, and there was no obvious change in the number of alleles when the sampling quantity increased (Figure 5A). At last, a total of 11 germplasms (12.64% of all ginkgo germplasms) that captured 100% of the detected diversity were set as core collections (Table S5). Furtherly, there were no significant differences in diversity indices, including Na, Ne, I, Ho, He, and PIC, between ginkgo germplasms based on pairwise comparisons between core collections and original germplasms using the Mann–Whitney U tests (Table S6), indicating that the core collections were representative of the original germplasms.

To further test if the core ginkgo collections represented the genetic diversity of all germplasms, PCoA analysis was performed to show the distribution of the original germplasms (87 individuals) and core collections (11 individuals) based on genetic diversity data (Figure 5B and Table S5). Most individuals from the core collections and original germplasms coincided in the middle part of the scatter plot with only a few exceptions, showing that the core collections were a good representation of the original germplasms (Figure 5B).

## 3. Discussion

To promote ginkgo breeding, it was necessary to conduct a comprehensive survey of the genetic diversity of ginkgo cultivars and clones at the genome-wide scale with InDel markers. Along with the advance of sequencing technology, InDels were widely used in genetic and genomic research for many advantages including high transferability and polymorphism, low cost of development, simple and efficient experimental procedure, and abundant distribution across the genome [28]. As a cost-effective sequencing technique, RAD-seq could simplify the complexity of the genome for InDel discovery and genotyping, which has been successfully used to mine InDel markers in different wood trees, such as poplar [22], tea [29], and citrus [23]. In this study, we identified a total of 279,534 InDel loci based on RAD-seq, which varied among different chromosomes, confirming that it was suitable for genome-wide marker development. In the ginkgo genome, increasing InDel size decreased the number of InDels detected. In addition, the most prevalent type was single-nucleotide Indels, similar to chickpea [12], soybean [30], and sesame [31]. Our InDel frequency was 27.30 per Mb (279.534 Indels in 10 Gb), lower than that obtained in other species [12,21]. RAD-seq is a restriction enzyme-guided sequencing approach that only targets part of the genome. In addition, to reduce the error rate of InDel identification, we focused on InDel markers with a length of no more than 10 bp. The false positive error rates would be increased with the length of InDels, which was susceptible to being influenced by read lengths, genome coverage and alignment methods [27]. In tomato, longer InDels do not always lead to more polymorphism as the polymorphism rate dropped to 43.3% when the InDel size was greater than 30 bp [32]. Therefore, these differences in the exploration of InDel variants may be due to the sequencing technique and bioinformatic parameters [26].

To better utilize ginkgo germplasms, the molecular diversity should be well-understood. However, previous genetic studies concentrated on wild ginkgo germplasms instead of cultivars and clones. The average PIC value of the 26 InDel markers was 0.517 in ginkgo cultivars and clones, lower than that of the SSR markers (0.781) used to identify genetic variation in ginkgo from ancient populations [14,16]. In our research, the average PIC value was more than 0.5 and the maximum value was 0.681, indicating that the developed InDels hold great potential for evaluating the genetic variation among different ginkgo germplasms. Generally, markers with PIC values of 0.5 or higher were extremely useful in distinguishing the polymorphic rate of a marker at a specific locus [33]. A total of 107 observed alleles were obtained in 87 accessions using 26 pairs of primers, with an average He value of 0.499. The genetic diversity index (H) values in ginkgo based on RAPD [34] and AFLP [15] were 0.191 and 0.3159, respectively. Based on our results, we determined a moderate level of genetic diversity in ginkgo. However, the average He value in our study was lower than that previously obtained with SSR markers (He = 0.808) [14]. Similarly, ginkgo had a lower level of genetic diversity than other gymnosperms evaluated using SSR markers such as *Taxus chinensis* (He = 0.261) [35] or *Abies fabri* (He = 0.739) [36], but similar to *Cupressus funebris* (He = 0.520) [37]. Indel markers are less polymorphic than codominant markers, such as SSR markers. Moreover, in our study, the samples were cultivars and clones, which completely differ from ginkgo germplasms mainly originating from wild populations. Generally, wild species preferred to own novel or specific alleles and diverse resources contributed to maintaining more sustainable biodiversity, but cultivated species or clones undergo natural and artificial selections within a limited number of superior genotypes [38]. Species domestication resulting from superior genotypes instead of undesirable genotypes leads to a reduction in inferior alleles over generations, which has a profound impact on the genes and genotypic frequencies of a population [39]. 

Ginkgo seeds are visually classified according to nut morphology and size and usually classified into four major cultivation groups, including “Changzi”, “Fozhi”, “Zhongzi”, and “Yuanzi” [40]. To our knowledge, morphological characteristics are extremely susceptible to environmental factors. Unfortunately, our cluster results were not consistent with traditional classifications based on seed morphological characteristics [40]. In other words, we found all ginkgo cultivars and clones showed an irregular distribution according to the structure, PCoA, and NJ analyses. There was an obvious controversy on the taxonomy of ginkgo when we compared the results from phenotypic characteristics and genotype differences. More ginkgo cultivars and clones were cultivated by artificial breeding and grafting in recent years for their vital economic and pharmacological value, which increased the difficulty to explore the accurate and efficient identification of different ginkgo germplasms with a limited number of markers. Therefore, the integration of phenotypic and genotypic information with more individuals belonging to a specific unit and more molecular markers covering the whole genome will avoid making misleading associations between phenotypes and genotypes.

Molecular marker-assisted breeding brings great challenges, opportunities, and prospects for conventional breeding [41]. Greater attention has been placed on core collections, which consisted of a minimal number of samples that represented the greatest genetic diversity. In our study, 11 ginkgo germplasms represented the core collections of the four major cultivation groups. Interestingly, the subset with a 12.64% sampling ratio yielded the largest allelic retention in ginkgo. Similar studies have reported allelic retention values of 99.5% and 95.74% in jujube [42] and pear [43], with sampling ratios of 15.6% and 24.2%, respectively. According to previous research, 5–20% of the sample size could encompass the genetic diversity of the entire collection [8]. Moreover, species diversity would be lost if they were used solely to determine the core collections with only limited molecular data or few germplasms without enough important morphological characteristics [18]. Therefore, for the ginkgo core collections, it was crucial to characterize valuable traits, such as the content of flavonoids and terpene lactones, and explore the genetic diversity with more germplasms.

## 4. Materials and Methods

### 4.1. Plant Materials 

All ginkgo germplasms used in this study were collected from the national ginkgo germplasm nursery of Pizhou (Pizhou City, Jiangsu, China) and the ginkgo germplasm resource nursery of Nanjing Forestry University (Nanjing City, Jiangsu, China), regarded as the largest centers of ginkgo germplasm collection, preservation, and utilization in China. The ginkgo cultivars and clones in these two nurseries have special agronomic traits, such as accumulating a high level of secondary metabolites (particularly for flavonoids and terpenoids) or varying widely in seed size. Nearly all were selected from wide or semi-wide germplasms and bred through grafting for more than 20 years. Young leaves of 87 different ginkgo cultivars and clones were collected from two nurseries and subsequently stored at −80 °C until further DNA extraction. Detailed information on each germplasm was summarized in Table S1. 

### 4.2. Library Preparation, and Sequencing 

According to the traditional classification criteria, ginkgo seeds were usually classified into four cultivation groups based on the morphology and size of nut, including “Changzi”, “Fozhi”, “Zhongzi”, and “Yuanzi” [40]. To better reveal the genetic diversity of ginkgo germplasms and develop polymorphic InDel markers, a total of 12 ginkgo cultivars from four cultivation groups (Table S1) were selected for genome sequencing. RAD sequencing library preparation was processed according to previous research [44]. Briefly, library preparation involved DNA digestion with *EcoR*I, P1 adapter/barcode ligation, DNA purification, size selection, P2 adapter/barcode ligation, and RAD tag amplification. Nearly 10 Gb raw data per sample were generated using paired-end sequencing with a read length of 150 bp based on the Illunina HiSeq 2500 platform (Majorbio Pharm Technology Co., Ltd., Shanghai, China). All raw sequence data were submitted to the National Center for Biotechnology Information database (BioProject ID PRJNA978007).

### 4.3. InDel Detection and Annotation 

The raw data from 12 ginkgo germplasms were processed with Stacks v 1.44 [45]. After raw sequence reads were demultiplexed, only reads with a clear *EcoR*I cutting site and the correct barcode were retained for further analysis. Adapter sequences and low-quality reads, including reads that have more than 10% nucleotides with a quality value lower than 30 (equals 0.1% sequencing error), were discarded. After trimming, we used BWA MEM software [46] to map reads to the ginkgo genome with default mapping parameters [16]. The HaplotypeCaller program in GATK v 3.8.0 [47] was used to call InDel variants across all samples simultaneously. Following the GATK best practices pipeline, variables were filtered using common hard filtering settings (QD < 2.0, FS > 200, ReadPosRankSum < −20, InbreedingCoef < −0.5, SQR > 10, maxIndelSize < 10). Lastly, variants with ≥70% call rate and sequence depth over 5 folds were retained. The distribution of InDels density in the ginkgo genomes of different ginkgo cultivars was investigated by generating density plots using rMVP [48] and shinyCircos v2.0 [49]. Using the ginkgo genome as a reference, InDel annotation was carried out using snpEff software [50]. InDels were categorized into intergenic regions, introns, or exons.

### 4.4. Primer Design and Experiment Validation 

Site-specific or single-copy primers were defined as those mapping to unique locations in the ginkgo genome, while primers that matched to numerous positions were disregarded. The annealing temperature was set to 55–60 °C and the length of primers to 18–22 bp. Overall, 100 pairs of InDel primers evenly distributed on 12 different chromosomes in the ginkgo genome were randomly selected to validate InDel primer accuracy and polymorphism levels in 12 ginkgo cultivars selected for RAD-seq. Only primers that effectively amplified and showed polymorphisms were chosen to evaluate the genetic diversity of all ginkgo germplasms. The polymorphic primers obtained after verification were mapped to chromosomes with MapChart software [51] to show their physical locations in the ginkgo genome. The InDel locus was amplified by PCR and all reactions were conducted in a 20 μL reaction mixture containing 50 ng genomic DNA, 10 μL 2 × Taq PCR Green MIX (Vazyme Biotechnology, Nanjing, China) and 0.1 μM of each primer pair. The PCR amplification procedures were as previously described and PCR products were separated using 6% polyacrylamide gel electrophoresis [52]. 

### 4.5. Genetic Diversity and Population Structure Analysis

A total of 26 InDel markers evenly distributed in the ginkgo genome showing 2–10 bp in silico fragment length polymorphism were selected to screen for polymorphism in 87 ginkgo cultivars and clones. The binary matrix generated with molecular data was converted to the required data format according to the instructions in GenAlEx v 6.5 [53]. The following genetic parameters—effective number of alleles (Ne), observed number of alleles (Na), expected heterozygosity (He), observed heterozygosity (Ho), Shannon’s information index (I), and the polymorphism information content (PIC)—were calculated using PowerMarker v 3.25 [54] and GenAlEx v 6.5. 

The population structure of 87 ginkgo germplasms was performed using the STRUCTURE v 3.0 [55] based on the individual-based Bayesian clustering method. To determine the optimal *K* value for different genotypes, 10 separate runs of a continuous series of *K* values from 1 to 10 were conducted. We performed 10 independent runs with 100,000 Markov chain Monte Carlo iterations after a burn-in of 100,000 steps to verify the consistency of the results. The optimal *K* was determined on STRUCTURE HARVESTER [56]. We processed these data to obtain the final results using DISTRUCT v 1.1 [57] and CLUMPP v 1.1.2 [58]. To further infer individual variation, the Nei’s genetic distance among all ginkgo germplasms were calculated in GenAlEx v 6.5, as an input for clustering analysis using principal component analysis (PCoA) and neighbor-joining (NJ) tree analysis with 1000 bootstrap replicates implemented in GenAlEx v 6.5 and MEGA v 7.0 [59], respectively. Bootstrap values more than 50 were listed on the dendrogram.

### 4.6. Core Germplasms Identification and Molecular Diversity Analysis

Based on the genetic diversity data, core collections were constructed using Core Finder v 1.1 [60], which is based on an M strategy with a Las Vegas-style random algorithm. During the building of the core collections, the strategy did not involve setting a sampling ratio because an appropriate sample ratio was automatically established. The genetic parameters (Na, Ne, Ho, He, I, and PIC) of core collections were calculated using GenAlEx v 6.5 and PowerMarker v 3.25. The PCoA analysis was conducted with GenAlEx v 6.5 to evaluate differences between the original germplasms and core collections.

## 5. Conclusions

To our knowledge, this is the first genome-wide investigation of InDels in ginkgo using RAD-seq that resulted in the development of a collection of useful polymorphic InDels. Of these, 26 InDel markers could divide the 87 ginkgo cultivars and clones into two obvious groups by population structure, PCoA, and NJ analyses. The ginkgo cultivars showed a moderate level of genetic diversity. A total of 11 core germplasms accounted for 12.64% of the initial germplasms successfully identified, which could be used for future breeding programs. The present findings will not only provide a useful resource for better germplasm utilization, facilitating the genetic improvement of ginkgo via marker-assisted breeding, but will also serve as a database for identification and traceability purposes. 

## Figures and Tables

**Table 1:** Genetic diversity analysis of ginkgo cultivars and clones based on the 26 pairs of InDel polymorphic markers.

Locus	(Na)	(Ne)	(I)	(Ho)	(He)	(PIC)
IND57	6	5.810	0.630	0.899	0.743	0.681
IND67	4	3.633	0.535	0.528	0.359	0.520
IND81	4	3.333	0.452	0.682	0.418	0.483
IND111	3	2.094	0.824	0.651	0.522	0.448
IND114	5	3.306	0.385	0.568	0.328	0.410
IND146	5	4.772	0.641	0.727	0.630	0.651
IND218	4	3.566	0.541	0.605	0.457	0.474
IND249	3	2.925	0.673	0.506	0.480	0.416
IND266	3	2.644	0.533	0.538	0.492	0.450
IND288	4	3.648	0.551	0.503	0.371	0.520
IND295	4	3.517	0.524	0.636	0.541	0.483
IND327	3	2.724	0.591	0.524	0.405	0.396
IND347	4	3.277	0.353	0.618	0.407	0.527
IND407	5	4.154	0.451	0.718	0.631	0.620
IND459	5	4.196	0.492	0.761	0.660	0.645
IND469	5	4.621	0.556	0.620	0.572	0.617
IND494	5	4.386	0.394	0.505	0.448	0.485
IND504	3	2.538	0.612	0.810	0.606	0.538
IND511	4	3.601	0.543	0.585	0.462	0.483
IND548	4	3.630	0.527	0.503	0.453	0.471
IND584	4	3.293	0.457	0.667	0.525	0.536
IND625	4	3.659	0.579	0.628	0.492	0.542
IND639	5	4.488	0.454	0.710	0.694	0.583
IND647	4	3.853	0.652	0.678	0.460	0.555
IND718	4	3.332	0.442	0.488	0.349	0.423
IND729	3	2.909	0.668	0.595	0.475	0.472
Mean	4.115	3.612	0.541	0.625	0.499	0.517

## Data Availability

The article contains all the information required to support its conclusions.

## Supplementary Materials

The following supporting information can be downloaded at: https://www.mdpi.com/article/10.3390/plants12132567/s1, Figure S1: The genome coverage of ginkgo by RAD-seq; Figure S2: Comparative distribution of the homozygosity and heterozygosity InDel among 12 ginkgo cultivars; Figure S3: Determination of the optimal number (K) for ginkgo cultivars and clones according to the Evanno’s admixture analysis; Table S1: The detailed information on ginkgo cultivars and clones in the study; Table S2: Summary of the sequencing quality of DNA libraries of 12 different ginkgo cultivars; Table S3: The information about insertions and deletions appeared simultaneously in at least six of 12 different ginkgo cultivars; Table S4: Detailed information of 26 pairs of InDel primers; Table S5: The description of individuals retained for the core ginkgo collections; Table S6: Mann-Whitney U Test (two-tailed) for the genetic diversity parameters of the core collections and the original germplasms.
