# Supplementary figures and images for: Molecular Characterization and Genetic Diversity of Ginkgo (Ginkgo biloba L.) Based on Insertions and Deletions (InDel) Markers
Source: Plants (Basel). 2023 Jul 6;12(13):2567. doi: 10.3390/plants12132567 (PMC10346307; doi:10.3390/plants12132567)

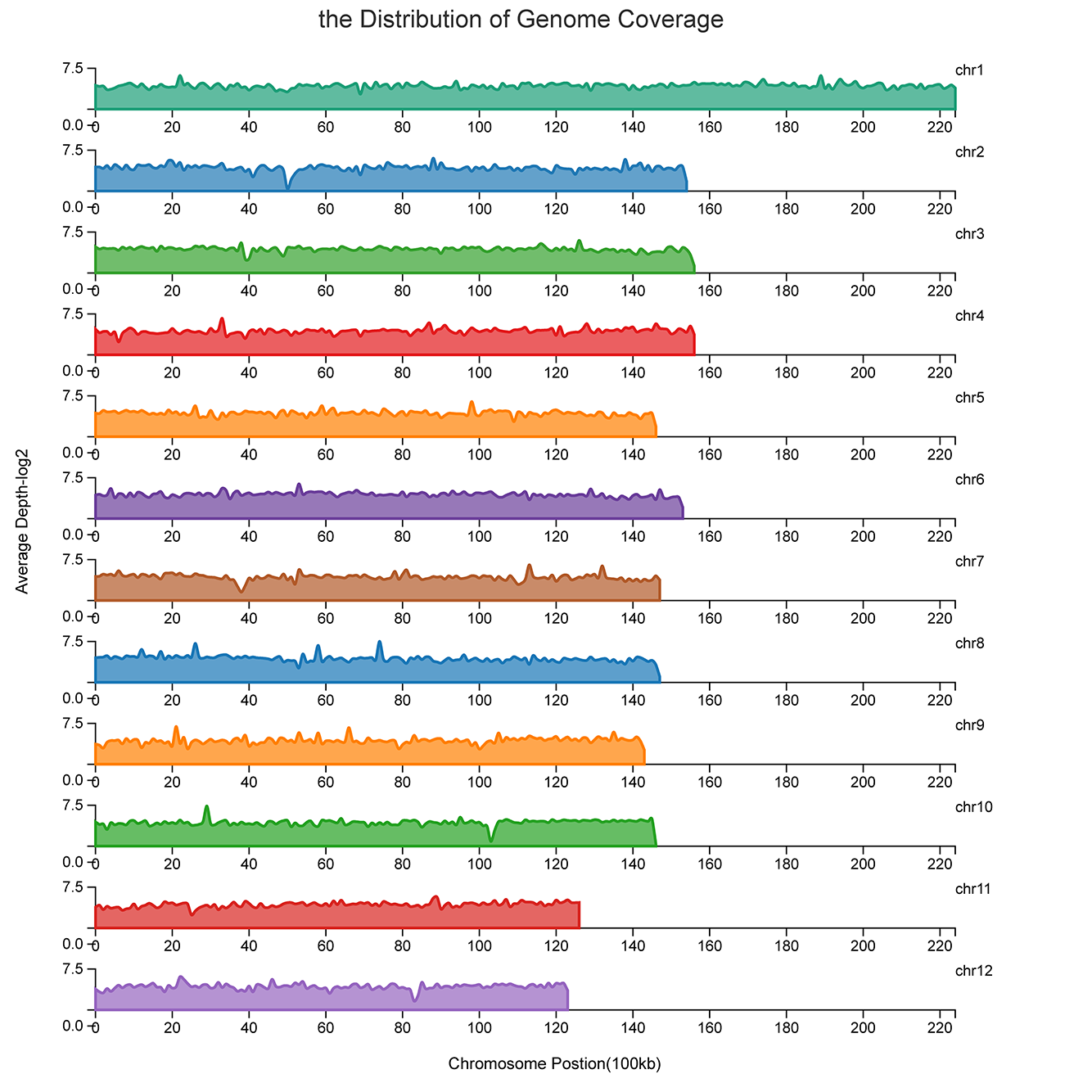

Supplement: Supplementary file 1 [file plants-12-02567-s001.zip › Figure S1.tif]

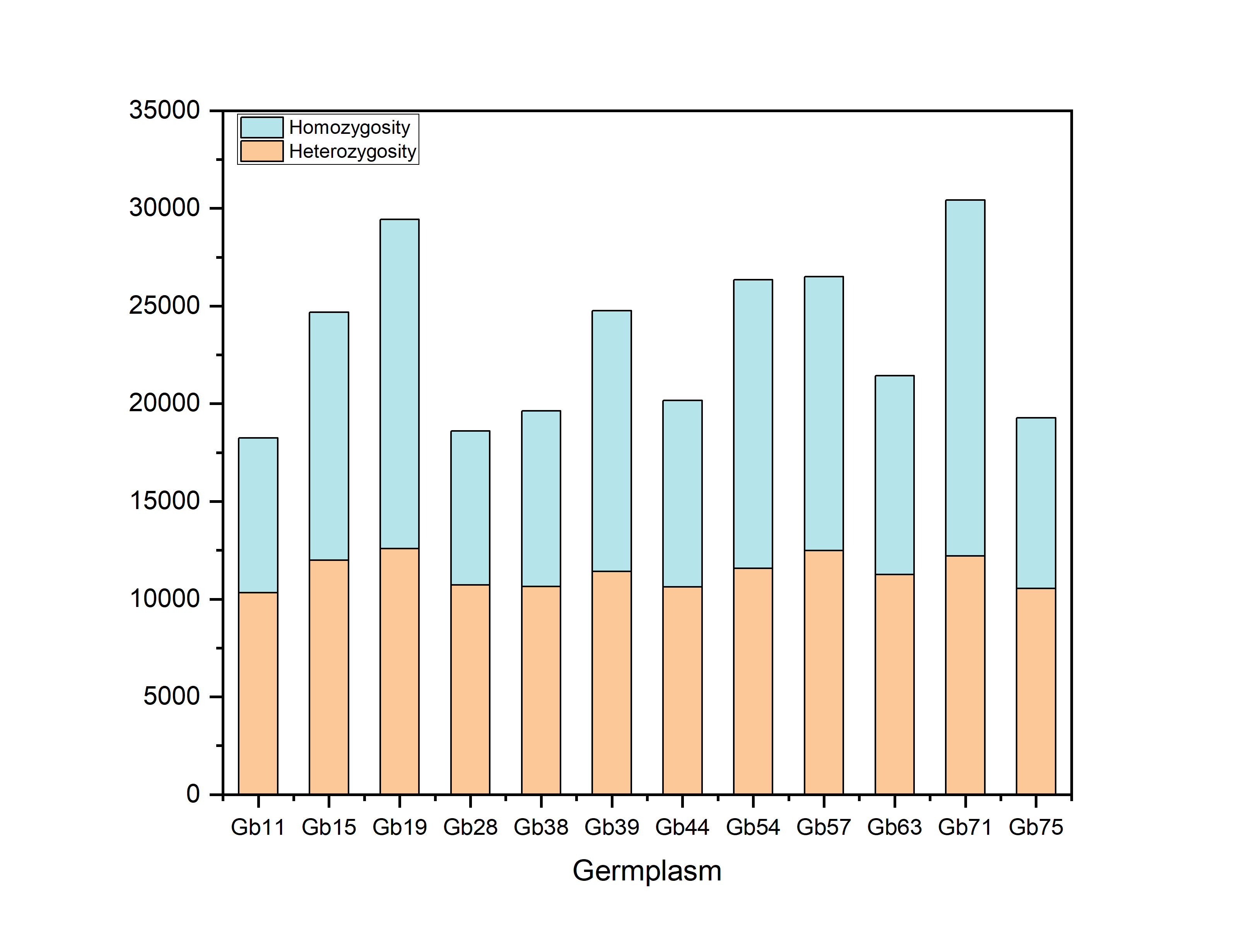

Supplement: Supplementary file 1 [file plants-12-02567-s001.zip › Figure S2.tif]

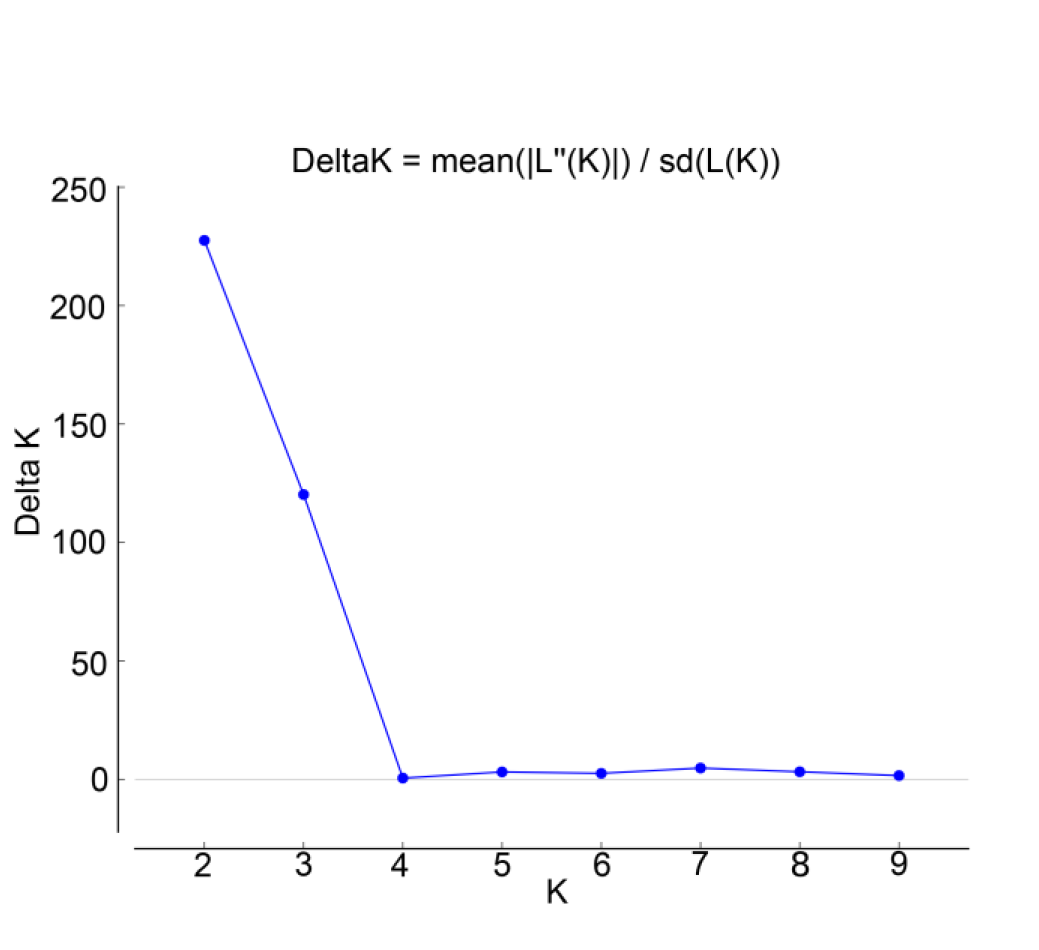

Supplement: Supplementary file 1 [file plants-12-02567-s001.zip › Figure S3.tif]
